# Effectiveness and possible brain mechanisms of cervical invasive vagus nerve stimulation (iVNS) intervention for avoidant/restrictive food intake disorder: a case report

**DOI:** 10.1093/psyrad/kkae016

**Published:** 2024-11-04

**Authors:** Suping Cai, Yihan Wang, Bofeng Zhao, Xiaoliang Li, Huan He, Kai Yuan, Qingchuan Zhao, Qinxian Huang, Bin Yang, Gang Ji

**Affiliations:** School of Life Sciences and Technology, Xidian University, Xi'an, Shaanxi 710071, PR China; School of Life Sciences and Technology, Xidian University, Xi'an, Shaanxi 710071, PR China; Xi'an International Medical Center Hospital, Northwest University, Xi'an, Shaanxi 710100, PR China; Xi'an International Medical Center Hospital, Northwest University, Xi'an, Shaanxi 710100, PR China; Xi'an International Medical Center Hospital, Northwest University, Xi'an, Shaanxi 710100, PR China; School of Life Sciences and Technology, Xidian University, Xi'an, Shaanxi 710071, PR China; National Clinical Research Center for Digestive Diseases and Xijing Hospital of Digestive Diseases, Xijing Hospital, Air Force Medical University, Xi'an, Shaanxi 710032, PR China; Xi'an International Medical Center Hospital, Northwest University, Xi'an, Shaanxi 710100, PR China; National Clinical Research Center for Digestive Diseases and Xijing Hospital of Digestive Diseases, Xijing Hospital, Air Force Medical University, Xi'an, Shaanxi 710032, PR China; National Clinical Research Center for Digestive Diseases and Xijing Hospital of Digestive Diseases, Xijing Hospital, Air Force Medical University, Xi'an, Shaanxi 710032, PR China

**Keywords:** avoidant/restrictive food intake disorder, invasive vagus nerve stimulation, brain magnetic resonance imaging, anxiety, depression, abdominal distension

## Abstract

**Background:**

We reported a case of cervical invasive vagus nerve stimulation (iVNS) treatment for avoidant/restrictive food intake disorder (ARFID) in a patient with severe anxiety and depression. This patient was even given a critical illness notice during his hospitalization and all treatment efforts were failed.

**Objective:**

We aimed to verfiy the effectiveness of iVNS in a patient with ARFID.

**Methods:**

We first attempted to perform cervical iVNS in this case and then observed the changes in clinical scores. We also analyzed the alterations in brain magnetic resonance imaging characteristics before and after iVNS using multi-modal neuroimagings.

**Results:**

After 18 days of iVNS (from 1 to 19 July 2023), the patient's clinical symptoms improved significantly and he rapidly gained 5 kg in weight. The brain functional characteristics of this patient tended toward those of the normal group. Functional connectivities of the medial of orbitalis prefrontal cortex returned to the normal range after iVNS.

**Conclusion:**

This is a precedent for performing cervical iVNS in an ARFID patient. Brain neural activity can be modulated through iVNS. The observed improvements in clinical scores and positive changes in brain function validated the effectiveness of iVNS. This case study provides evidence that this intervention technique could be used to reduce the burden on more similar ARFID patients.

## Introduction

Zhou (pseudonym), male, 34 years old and height of 170 cm, complained of difficulty feeding and defecating, nausea, vomiting, and abdominal distension for 15 years without obvious inducement. In recent years, Zhou's anxiety and depression symptoms and food intake disorder has worsened. He sought help from many gastrointestinal and psychosomatic hospitals and underwent all the relevant tests. Zhou was diagnosed with avoidant/restrictive food intake disorder (ARFID), refractory constipation accompanied by anxiety, and depression. He was treated with oral psychotropic drugs, Chinese herbs, acupuncture, moxibustion, and other treatments. However, all efforts failed. His weight still decreased from 50 to 31 kg. His symptoms seriously affected his life. He was even given a critical illness notice during his hospitalization.

The vagus nerve is a crucial bond between the central nervous system and somatic organs. It plays a crucial role in memories, emotions, and pain (Wang *et al*., 2021). Vagus nerve stimulation (VNS) approved by FDA as an alternative intervention tool for neuropsychiatric diseases, such as depression (Rong *et al*., 2014; Rush *et al*., 2000), and has been shown to substantially improve the clinical performances (Wang *et al*., 2021; Rong *et al*., 2016). However, there is no precedent for invasive VNS (iVNS) in ARFID patients (or in patients with specific symptoms such as in this case of Zhou). After considering Zhou's clinical symptoms and obtaining ethical approval, we attempted to implant a VNS device in Zhou's neck in the hope of improving his clinical symptoms. However, the patient's weight and current physical condition were insufficient to support an invasive implantation procedure, and nutritional supplementation was required prior to iVNS. After 3 months of consecutive hospital treatment and nutritional supplementation, his weight subsequently increased from 31 to 40 kg. Importantly, the 3-month weight gain was supported by the hospital nutritional fluids, however, the patient needed to work and lead a normal life, and the support from the hospital nutritional fluids was only temporary. A long-term supportive treatment programme was urgently needed.

## Methods and materials

### Ethics statement

We confirm that all experimental protocols were approved by the ethics committee of the Affiliated Hospital of the Fourth Military Medical University in China, in which the ethical standards of the institutional and national research committee are in accordance with the 1964 Helsinki declaration and its later amendments. Written informed consent was obtained from Zhou and the other participants in the healthy control (HC) group, or surrogates.

### Clinical data

Zhou was a 34-year-old male, height 170 cm, and weighed 31 kg when he first came to the hospital. Zhou's psychotic symptoms were assessed using the self-rating anxiety scale (SAS), self-rating depression scale (SDS), and self-reported inventory (SCL-90). During his hospitalization, he was given a critical illness notice. After three consecutive months of in-patient treatment, nutritional supplementation increased his weight from 31 to 40 kg. The day before VNS was initiated, his weight was 40 kg. Thirty-seven age-matched (20–45 years, mean age 34.6 years) HCs with no mental or physical conditions were selected for comparative analysis.

### Implanted VNS parameters

The iVNS equipment (model G112, 42 × 36 × 6.8 mm, weight 14 g, Beijing Pins Medical Co., Ltd in China) was implanted on 1 July 2023. The stimulus parameters were as follows: bilateral (left), intermittent stimulus, amplitude = 0.2 mA, pulse width = 250 μs, frequency = 10 Hz, stimulation time = 60 s with an interval of 30 minutes; impedance = 2–8 kΩ; magnet mode: amplitude = 0 mA, pulse width = 500 μs, frequency = 10 Hz, stimulation time = 60 s. These parameters did not change during the 18 days (the observation period of the current experiment) of stimulation.

### T1-weighted magnetic resonance imaging (MRI) data acquisition pre- and post- iVNS

All images acquired by MRI were scanned using a 3T Philips Medical Systems Nederland B.V. scanner. Structural MRI images were obtained using a high-resolution T1-weighted magnetization-prepared rapid gradient echo sequence. The parameters were as follows: echo time (TE) = 11 ms, repetition time (TR) = 0.7 s, flip angle (FA) = 7°, FOV = 256 × 256 mm, matrix = 256 × 256, slice thickness = 1 mm, inversion time = 1100 ms, and 192 coronal slices. After 18 days of iVNS treatment, T1-MRI images were collected again using the same scanning parameters. The iVNS device was turned off when the images were acquired from the patient; therefore, all magnetic resonance images were resting-state images [similar to functional MRI (fMRI) and diffusion tensor imaging (DTI) images].

### Resting-state functional MRI (fMRI) acquisition

The parameters of the fMRI data were as follows: TE = 30 ms, TR = 2 s, FA = 90°, slice thickness = 3.0 mm, slices = 50, time points = 210, matrix size = 64 × 64, and voxel size = 3 × 3 × 3 mm. After 18 days of iVNS treatment, fMRI images were collected again using the same scanning parameters.

### DTI acquisition

The DTI data of this patient of pre-iVNS was not scanned. We only obtained the DTI data of post-iVNS. The parameters are as follows: *b* = 1000 s/mm^2^, TE = 90 ms, TR = 7500 ms, FA = 90°, FOV = 256 × 256 mm, matrix = 384 × 384, slice thickness = 2 mm, and 60 slices.

### T1-weighted MRI processing and analysis

T1-weighted MRI processing and analysis were performed using the FSL protocol with the FMRIB Software Library v.4.1 (Fischl, 2012). We extracted the cortex thickness, volume, and area measurements of Zhou and 37 HCs. Then, 2* sigma principle, we chose the mean value minus 2* standard deviation (mean-2*SD) as the lower limit and the mean + 2*SD as the upper limit in HCs. If a value of pre- or post-iVNS was not within this interval, it was considered abnormal.

### fMRI processing and analysis

fMRI image processing was addressed using a MATLAB toolbox called DPABI (Yan *et al*., 2016). There were eight steps: removing the first 10 time points, slice timing correction, correcting for head motion, normalizing to individual anatomical images, smoothing images, removing linear trends, filtering (0.01–0.1 Hz), and regressing the covariates. We analyzed pre- and post-iVNS fMRI data using ALFF (amplitude of low frequency fluctuation) (Yang *et al*., 2007), ReHo (regional homogeneity) (Zang *et al*., 2004), and FC (functional connectivity) (Rogers *et al*., 2007) methods. ALFF is a neuroimaging measure of the level of spontaneous neural activity in different brain areas. It is calculated by analyzing the amplitude of signal fluctuations in a specific frequency range (0.01–0.1 Hz). ReHo has emerged as a key method for investigating functional connectivity in the brain by assessing the temporal synchrony of regional neural activity. This technique involves calculating the degree of similarity, or coherence, in blood oxygen level-dependent signal fluctuations within a given region of interest, typically defined by a small cluster of adjacent voxels. By using ReHo, researchers can uncover coordinated patterns of activity at the micro level that reflect the operating principles of neural networks, providing insights into both healthy and pathological brain states. For the ALFF and ReHo, we aimed to analyze changes in whole-brain measures of function before and after iVNS. The ReHo/ALFF differences between the pre-iVNS and HC groups, and the changes between the post-iVNS and HC groups were evaluated by determining whether the values for that patient were within the interval (mean-2*SD, mean + 2*SD) of the HCs, respectively. For the FC analysis, we selected 139 related brain regions (trying to cover the whole brain) from the AAL3 template to calculate 9591 ($C_{139}^2$) functional connections. If the FC value of pre- or post-iVNS was not within this interval (mean-2*SD, mean + 2*SD), it was considered abnormal.

Head movement may have an affect on resting-state fMRI measurements, such as the FC method (Power *et al*., 2015). To investigate whether the head motion value of this patient was within the interval (mean-2*SD, mean + 2*SD) of that of the HCs, we extracted the head movement information of each participant in this case and that of HCs. We observed whether the head movement value in this case was within the normal group interval range.

### DTI processing and analysis

Following DTI acquisition, eddy current and motion correction were performed using a 12 degrees of freedom affine transformation using FSL (FMRIB) (Smith *et al*., 2006). The resulting registration matrices for each DTI volume were used to update the b-matrices prior to calculation of the diffusion tensor. The data was then imported into the Analysis of Functional NeuroImages software package. Eigenvalues and eigenvectors were extracted and the fractional anisotropy calculated. The DTI data of the pre-iVNS was not scanned. Therefore, in this case report, we only compared the fractional anisotropy value of this patient of post-iVNS with those of the HC group.

## Results

### Demographic and clinical results

**Figure 1:**
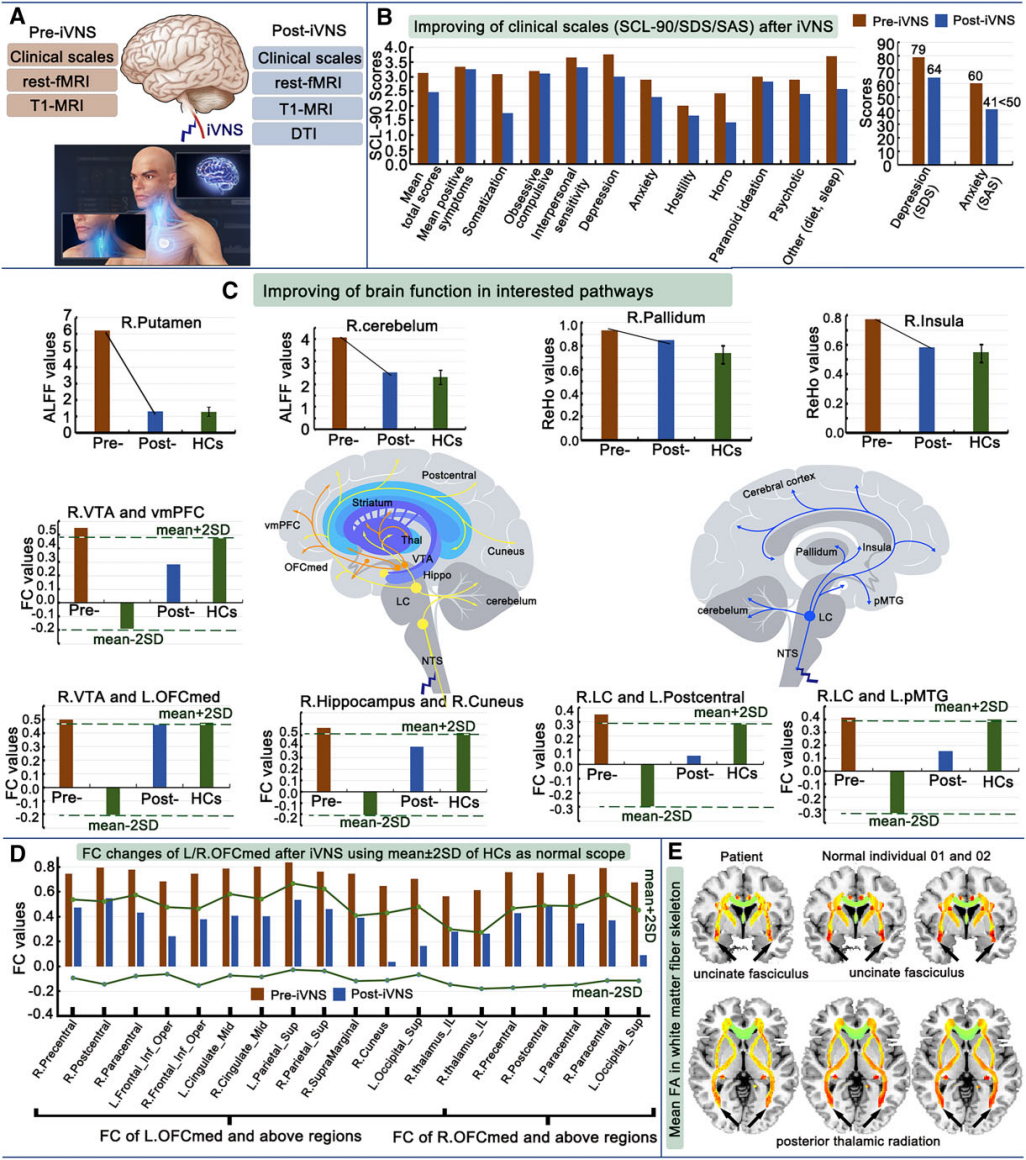
Changes in clinical scales and brain MRI in ARFID treated with cervical iVNS. (**A**) Experimental procedure. (**B**) Comparison of clinical scales between pre- and post-iVNS. (**C**) Improvements in brain function of interested pathways among HCs, pre- and post-iVNS using bar graphs. (**D**) FC changes of L/R.OFCmed between pre- and post-iVNS using the mean ± 2SD of HCs as normal ranges. (**E**) Intuitive comparison of mean fractional anisotropy in the white matter fiber skeleton between post-iVNS individual and those of two normal individuals. All abbreviations used in this figure are the same as those used in the main text.

After 18 days of iVNS (from 1 to 19 July 2023) (Fig. 1A), the patient SCL-90 and SDS scores improved, and his SAS score returned to the normal range (Fig. 1B and Table 1). Zhou reported that his anxiety symptoms had disappeared. His weight increased from 40 kg (pre-iVNS) to 45 kg. He was able to socialize normally, such as going out with his family instead of staying indoors and speaking little. His diet and sleep have gradually improved and his constipation symptoms improved. The head movement of this patient was within the normal group interval range (Table 1).

**Table 1:** All demographic information.

	Mean scores of the patient		Reference interval/reference (mean ± SD)	Changes after iVNS treatment
Clinical scale	Pre-iVNS	Post-iVNS		
**Weight (kg)**	40	45	61–63	weight gain
**SCL-90 Scale**				
Mean total scores	3.13	2.48	1.44 ± 0.43	score down
Positive items	82	60	24.92 ± 18.41	items decreased
Negative items	8	30	65.08 ± 18.33	items increased
Mean positive symptoms	3.34	3.25	2.60 ± 0.59	score down
Somatization	3.08	1.75	1.37 ± 0.48	moderate to no
Obsessive–compulsive symptoms	3.2	3.1	1.62 ± 0.58	moderate to moderate
Interpersonal sensitivity	3.67	3.33	1.65 ± 0.51	moderate to moderate
Depression	3.77	3	1.50 ± 0.59	moderate to moderate
Anxiety	2.9	2.3	1.39 ± 0.43	mild to mild
Hostility	2	1.67	1.48 ± 0.56	mild to no
Panic behavior	2.43	1.43	1.23 ± 0.41	mild to no
Paranoid ideation	3	2.83	1.43 ± 0.57	moderate to mild
Psychotic symptoms	2.9	2.4	1.29 ± 0.42	mild to mild
Diet, sleep	3.71	2.57	–	moderate to mild
**Depression (SDS)**	79	64	<53	score down
**Anxiety (SAS)**	60	41	<50	mild to no
**Head motion**	**0.142**	**0.144**	**(0.109, 0.145)**	**in normal range**
**HCs**	**37 males**	a**ge (20–45)**	**mean age 34.6**	**all are normal**

### Changes in the mean ALFF after iVNS

**Figure 2:**
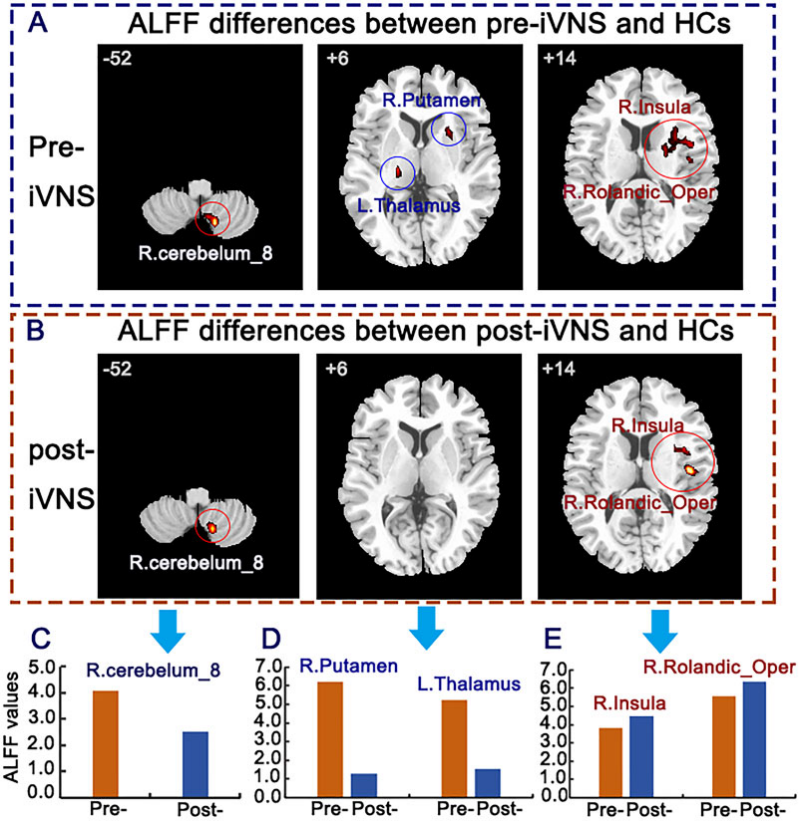
ALFF differences between pre-iVNS, post-iVNS, and HCs. (**A**) ALFF differences between pre-iVNS and HCs. (**B**) ALFF differences between post-iVNS and HCs. (**C**–**E**) Bar scale images of the intuitive differences in the right cerebellum_8, right putamen, left thalamus, right insula, and right opercular part of the Rolandic region.

We compared the mean ALFF values before and after iVNS stimulation with those of HC group. The main results are shown in Figs 1C and 2. The ALFF values in the right cerebellum_8, right putamen and left thalamus are decreased after iVNS. The ALFF values in the right insula and right rolandic operculum are increased after iVNS.

### Changes in the mean ReHo after iVNS

**Figure 3:**
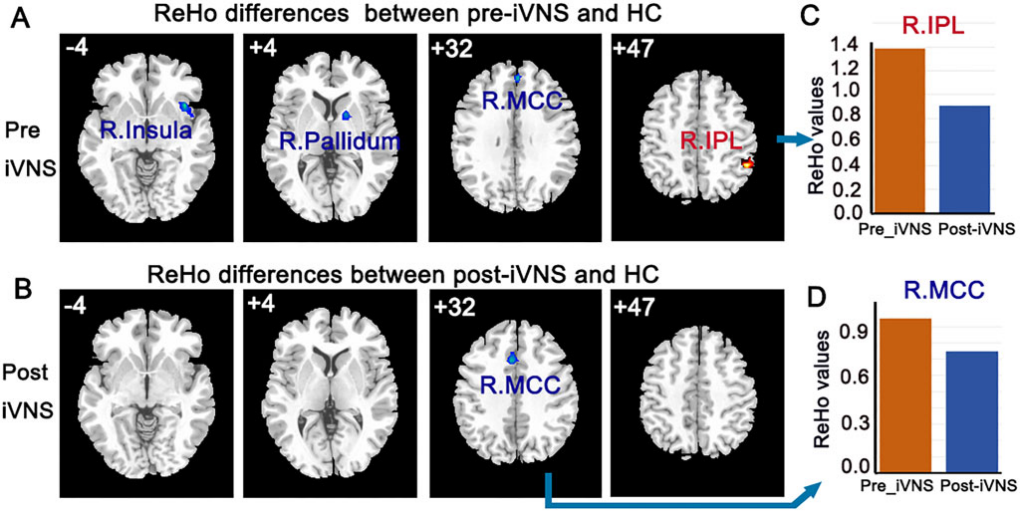
ReHo differences between the pre-iVNS and post-iVNS patient and HCs. (**A**) ReHo differences between pre-iVNS and HCs. (**B**) ReHo differences between post-iVNS and HCs. (**C, D**) The intuitive differences in the bar graphs of the right inferior parietal lobule (R.IPL) and right middle cingulate gyrus (R.MCC) are shown.

We compared the mean ReHo before and after iVNS stimulation with that in the HC group, respectively. The main results are shown in Figs. 1C and 3. The ReHo values in the right insula, right pallidum, right middle cingulate gyrus, and right inferior parietal lobule were reduced after iVNS. Intuitively, we found that the ReHo values improved after iVNS treatment.

### Changes in functional connectivity after iVNS

In total, 9591 ($C_{139}^2$) functional connections (FCs) are obtained in the patient of pre-iVNS and post-iVNS and in HCs. On the basis of on the 2*sigma principle and the mean FC values in HCs, there are 203 out-of-scope (mean-2*SD, mean + 2*SD) FCs in Zhou of pre-iVNS. We first focused on the norepinephrine (NE), serotonin (5-HT), and dopamine (DA) pathways. The reasons for choosing these three pathways are explained in the discussion section. We wanted to investigate which connections in the three pathways changed after iVNS. The main results are shown in Fig. 1C. Of the 203 FCs, 27 were within the scope (mean-2*SD, mean + 2*SD) after iVNS. Interestingly, these 27 FCs were mainly connections related to the orbitofrontal cortex (OFCmed) (Fig. 1D).

### Changes in structural measures and fractional anisotropy after iVNS

Structural measures of cortical thickness, volume, and area in the patient have no measures out of range (mean-2*SD, mean + 2*SD) both in pre- and post-iVNS. The mean fractional anisotropy of the white matter fiber skeleton after iVNS of this patient was different from the HC individuals. Figure 1E is an example of an intuitive comparison. The posterior thalamic radiating fiber and uncinate fasciculus showed obvious incompleteness in patient Zhou (fractional anisotropy maps of the three individuals were randomly selected).

## Discussion

There is no precedent for iVNS in patients with restrictive food intake disorder or in patients with specific symptoms such as the current case. This is the first attempt to perform cervical iVNS in this case. The observed improvements in clinical scores and positive changes in brain function validate the efficacy of iVNS. This case study gives us great confidence to reduce the burden on more similar cases. Importantly, we sought to explain the possible mechanism underlying these positive changes from iVNS using multimodal neuroimaging.

To facilitate the interpretation of the changes in FCs, we focused on three major neurotransmitter pathways (NE, 5-HT, and DA pathways) that have been largely identified in the literature. These neurotransmitters project to most of the brain regions involved in emotion and food intake, or are present in a single brain region. We simply wanted to observe the effects of vagus stimulation on the functional connectivity of the brain regions of interest. Alterations in functional brain activity were observed in the three pathways involved in feeding and emotions, including mood regulation, reward mechanisms, and anxiety and stress responses. These changes have been linked to a range of mental health conditions, including anxiety, depression, and eating disorders (Furmaga *et al*., 2011; Wang *et al*., 2021). ARFID is not primarily motivated by concerns about body image or fear of weight gain. Instead, it often involves underlying psychological and neurobiological factors that are influenced by changing the brain's neurotransmitter systems. This patient showed different degrees of functional recovery after iVNS in different areas. With respect to the DA pathway, the ALFF value of the putamen was decreased in the patient after iVNS, and tended to be similar to that of HCs. The FC between the right ventral tegmental area (VTA) and ventromedial prefrontal lobe and the FC between the right VTA and left medial of the orbitalis prefrontal cortex returned to their normal ranges. For the 5-HT pathway, the ALFF value of the cerebellum was decreased after iVNS, and tended towards that of the HCs. The FC between the right hippocampus and cuneus had returned to its normal range. For the NE pathway, the ReHo values of the insula and pallidum and the FC between the right locus coeruleus and left postcentral, and the FC between the right locus coeruleus and left pole of the middle temporal gyrus returned to their normal ranges. These results suggest that brain stimulation via the vagus nerve may be able to rewire and repair damaged neural connections or spontaneous neural activity.

Interestingly, the FCs of pre-iVNS that exceeded the threshold (mean ± 2*SD) were mainly concentrated in the right and left medial of the orbitalis prefrontal cortex, and all of these FCs returned to their normal ranges after 18 days of iVNS. Neural circuits involving the OFCmed are heavily modulated by dopaminergic signaling pathways, particularly those associated with the nucleus accumbens and the VTA, which are vital to reward processing. Studies utilizing fMRI have shown heightened activation in the OFCmed when participants engage with food-related stimuli, highlighting its central role in evaluating the desirability of food items. Furthermore, the OFCmed is involved in inhibitory control and emotional regulation, enabling individuals to modulate their responses to food based on contextual cues, personal goals, and memory. The VTA is a major producer of DA. The connection between the VTA and the prefrontal cortex is one of the major dopamine DA pathways. For this patient, the OFCmed and ventromedial prefrontal lobe were the main areas that showed positive changes after iVNS. iVNS reduced the excessive control of the prefrontal cortex over subcortical regions, resulting in better clinical outcomes. The LC is a key region for projecting signals from the vagus nerve to the relevant cortical regions. In this patient, these relevant response regions may be the left postcentral and left pole of the middle temporal gyrus.

The reduced ALFF and ReHo values observed in the putamen, cerebellum, insula, and pallidum after iVNS may indicate good responses of these regions. These values tend to be close to the average values measured in the HCs. iVNS can reduce the abnormal spontaneous activity of these regions, which are loosely associated with human feeding behavior via the processing of external stimuli (Kantonen *et al*., 2021). In addition, iVNS may work quickly by modifying the FCs of the OFCmed (Wang *et al*., 2021). The OFCmed should receive more attention in follow-up studies (Heather Hsu *et al*., 2020). It is important to gain a deeper and more nuanced understanding of the functioning of these regions and others that are implicated in feeding, depression and anxiety through their altered functional activities (Kenwood *et al*., 2022; Yin *et al*., 2021; Rolls *et al*., 2020). For DTI, the eigenvalues and eigenvectors were extracted and the mean fractional anisotropy was calculated. Unfortunately, no pre-iVNS DTI images were acquired. Therefore, we compared the fractional anisotropy values after iVNS with those of the HCs. We observed lingering differences in the fractional anisotropy of the uncinate fasciculus and posterior thalamic regions. The uncinate fasciculus is a white matter tract in the human brain that connects the anterior temporal lobe with the OFC and other regions involved in emotional regulation and reward processing. The posterior thalamic region is primarily involved in influencing how individuals respond to environmental stimuli, including those related to hunger and satiety. These regions facilitate the acute processing of sensory information, allowing for timely and adaptive responses to food availability and satiation cues. These two white matter regions did not return to normal fractional anisotropy values after iVNS stimulation, suggesting that white matter recovery may be slower and require a longer follow-up.

In conclusion, we reported an ARFID case of cervical iVNS treatment and described the changes in clinical scores and brain function before and after iVNS. This is a precedent for performing cervical iVNS in the ARFID patient. Neural activity in the brain could be modulated by iVNS. The observed improvement in clinical scores and positive changes in brain function validated the efficacy of iVNS. This case study provides great confidence in the use of this intervention technique to reduce the burden on more ARFID patients.

The limitations of the current work should be discussed here. This was a single-case study, although we included 37 normal controls for comparative analysis. We will continue to follow the case longitudinally. A DTI image of the pre-iVNS of the current case was not acquired. Thus, we did not compare the changes in the white matter indices before and after iVNS.

## Data, materials, and code availability

Send an email to Qinxian Huang (Qinxianhuang2022@163.com) to obtain freely.
